# Supplementary figures and images for: Lactobacillusjohnsonii L531 Protects against Salmonella Infantis-Induced Intestinal Damage by Regulating the NOD Activation, Endoplasmic Reticulum Stress, and Autophagy
Source: Int J Mol Sci. 2022 Sep 8;23(18):10395. doi: 10.3390/ijms231810395 (PMC9499332; doi:10.3390/ijms231810395)

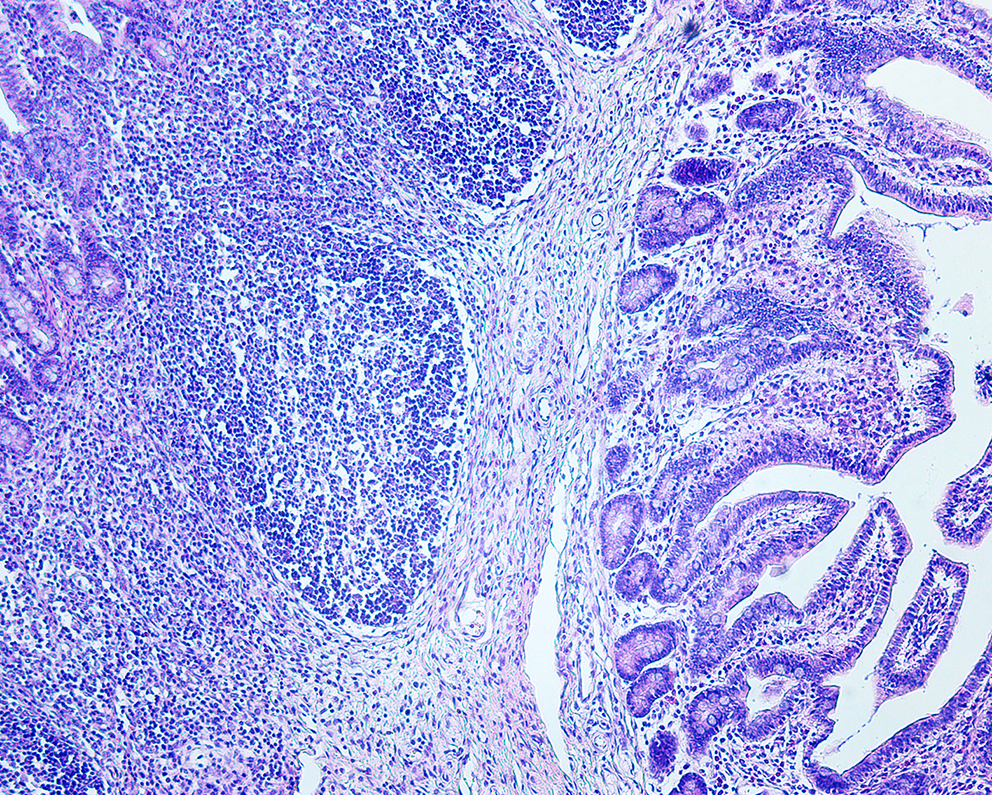

Supplement: Supplementary file 1 [file ijms-23-10395-s001.zip › ijms-1877568-supplementary/Original Date/Ileum-CN-down.tif]

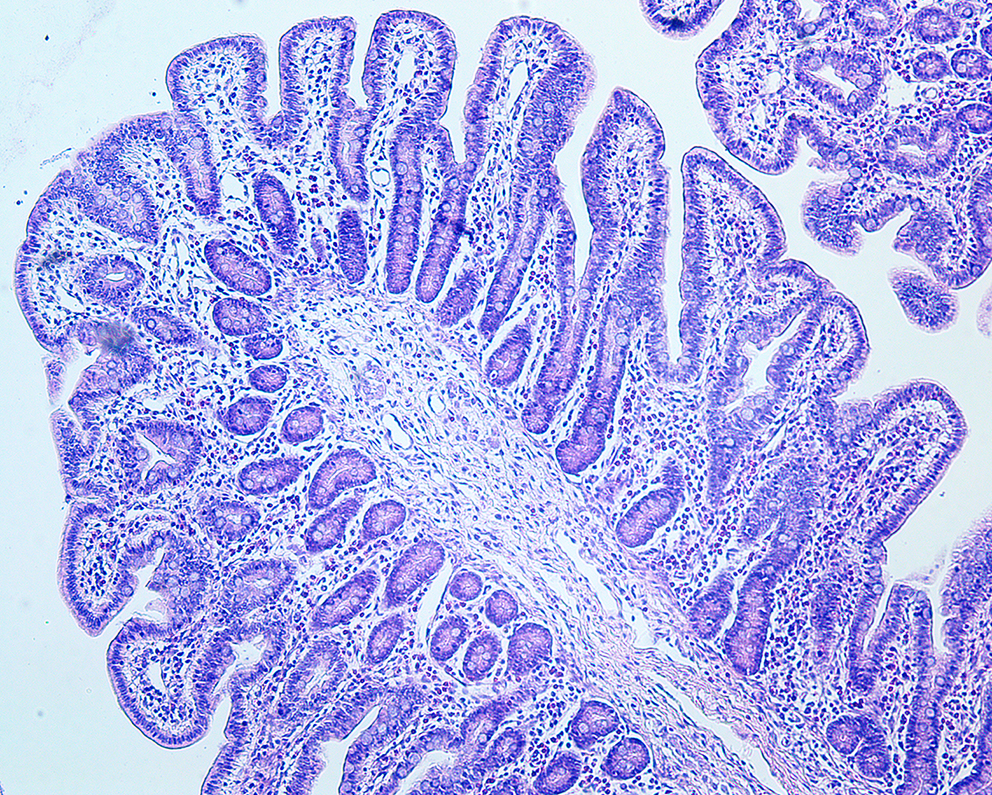

Supplement: Supplementary file 1 [file ijms-23-10395-s001.zip › ijms-1877568-supplementary/Original Date/Ileum-CN-up.tif]

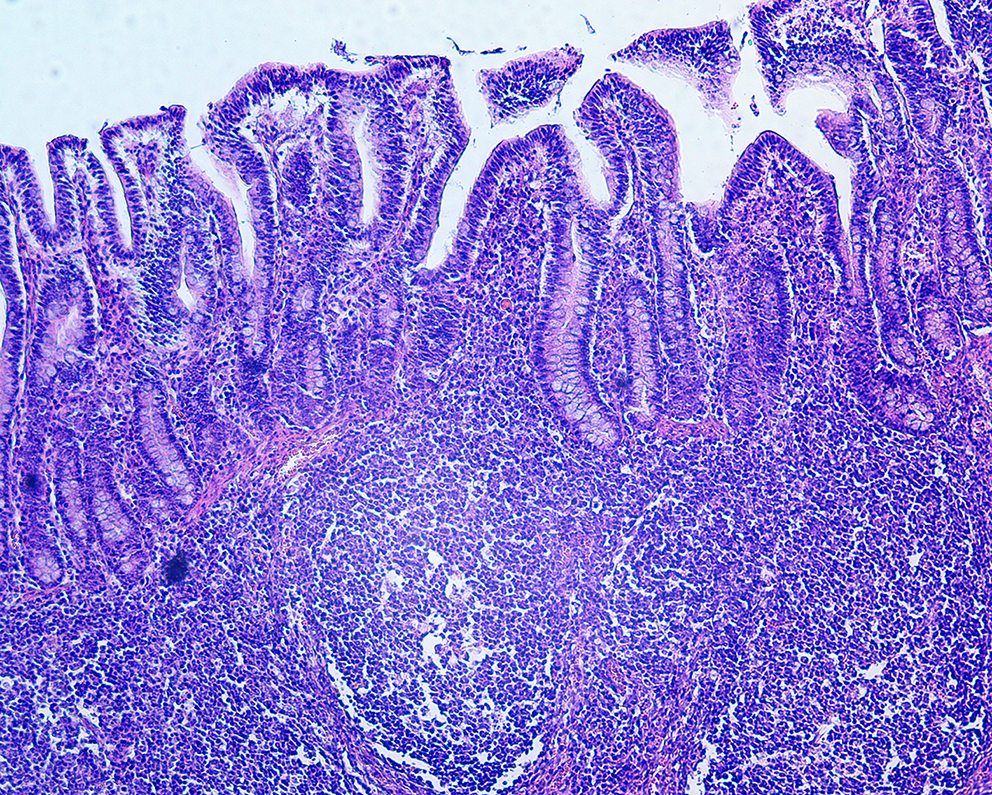

Supplement: Supplementary file 1 [file ijms-23-10395-s001.zip › ijms-1877568-supplementary/Original Date/Ileum-LS-down.tif]

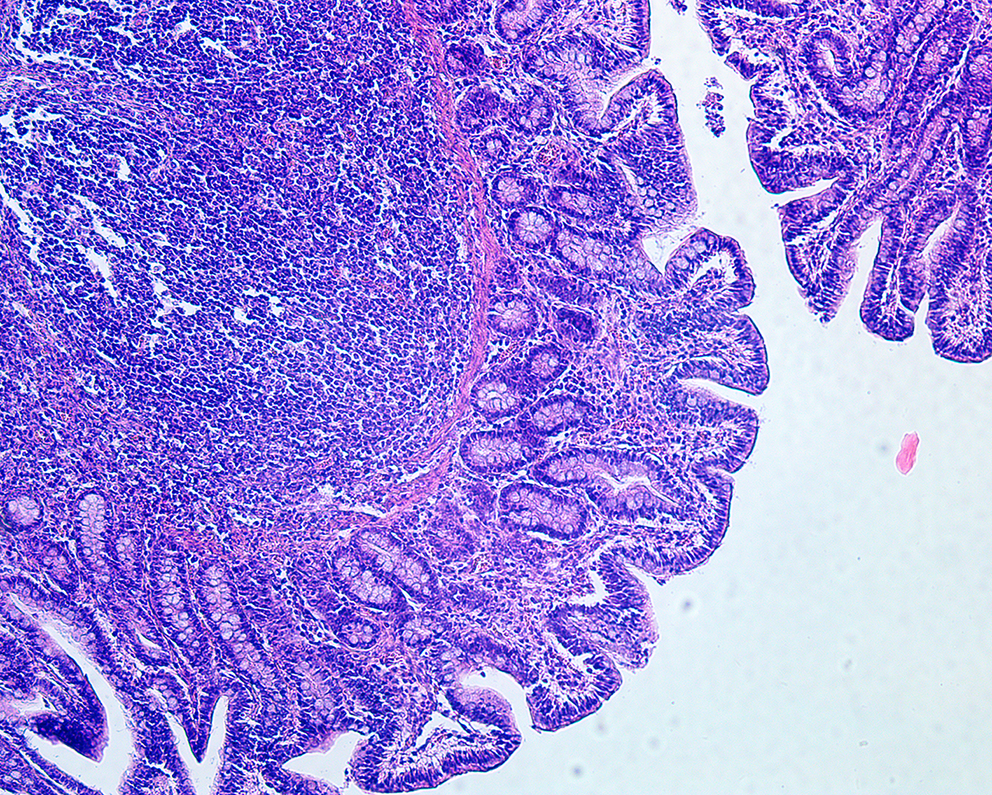

Supplement: Supplementary file 1 [file ijms-23-10395-s001.zip › ijms-1877568-supplementary/Original Date/Ileum-LS-up.tif]

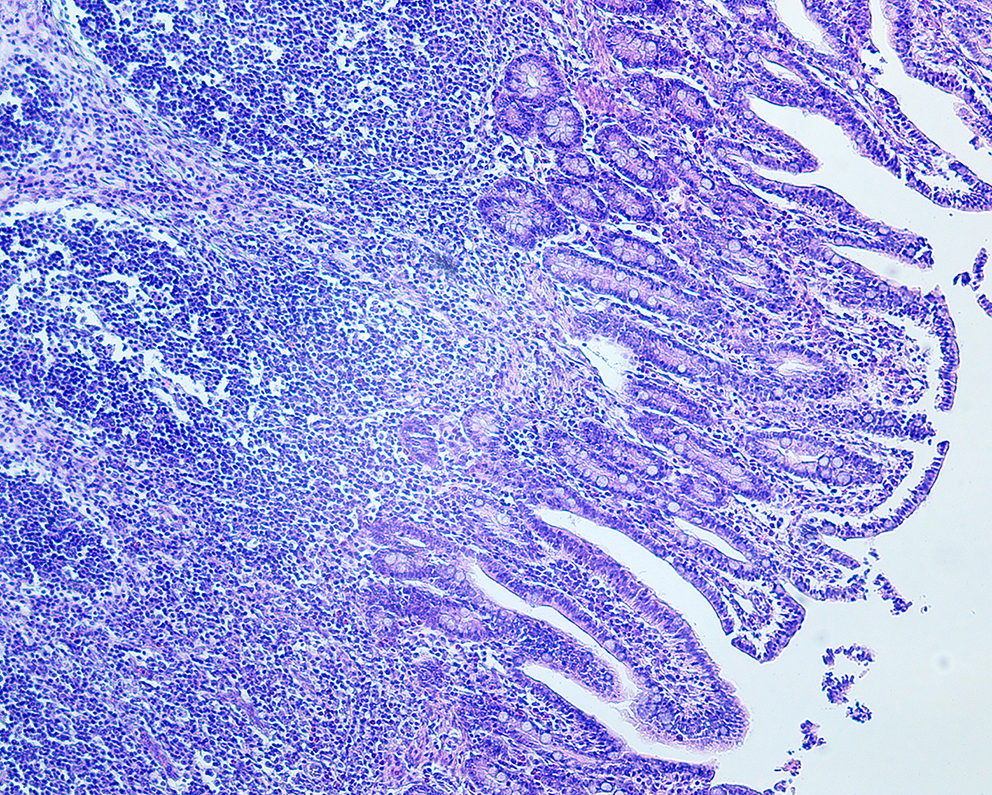

Supplement: Supplementary file 1 [file ijms-23-10395-s001.zip › ijms-1877568-supplementary/Original Date/Ileum-SI-down.tif]

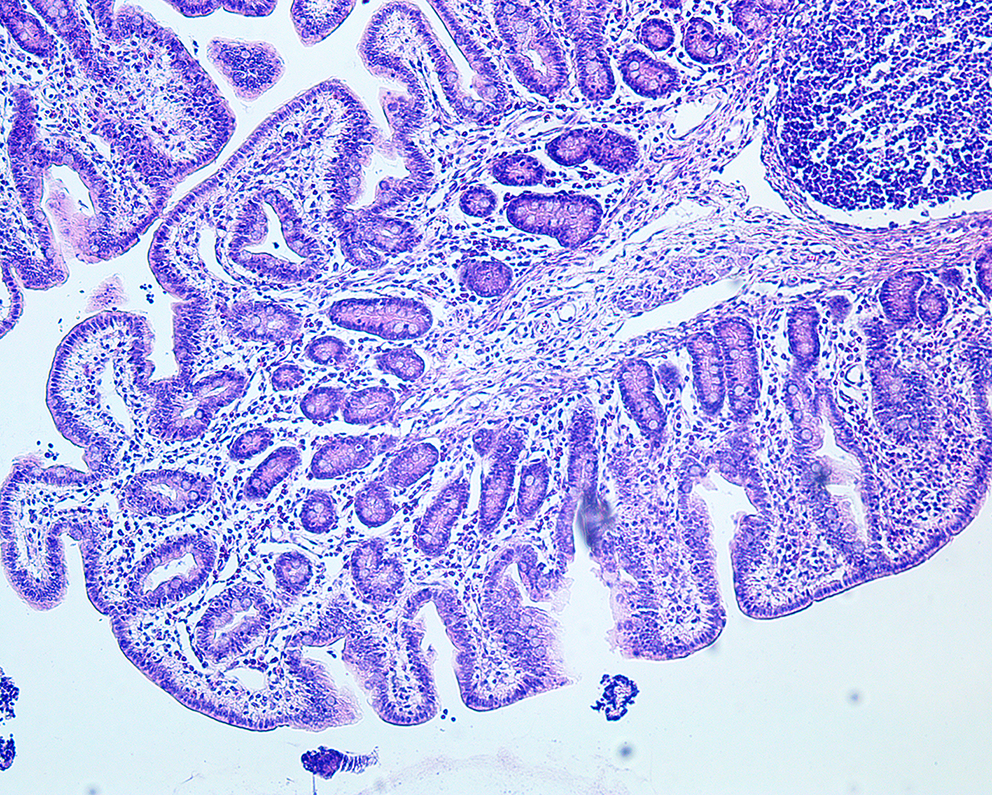

Supplement: Supplementary file 1 [file ijms-23-10395-s001.zip › ijms-1877568-supplementary/Original Date/Ileum-SI-up.tif]

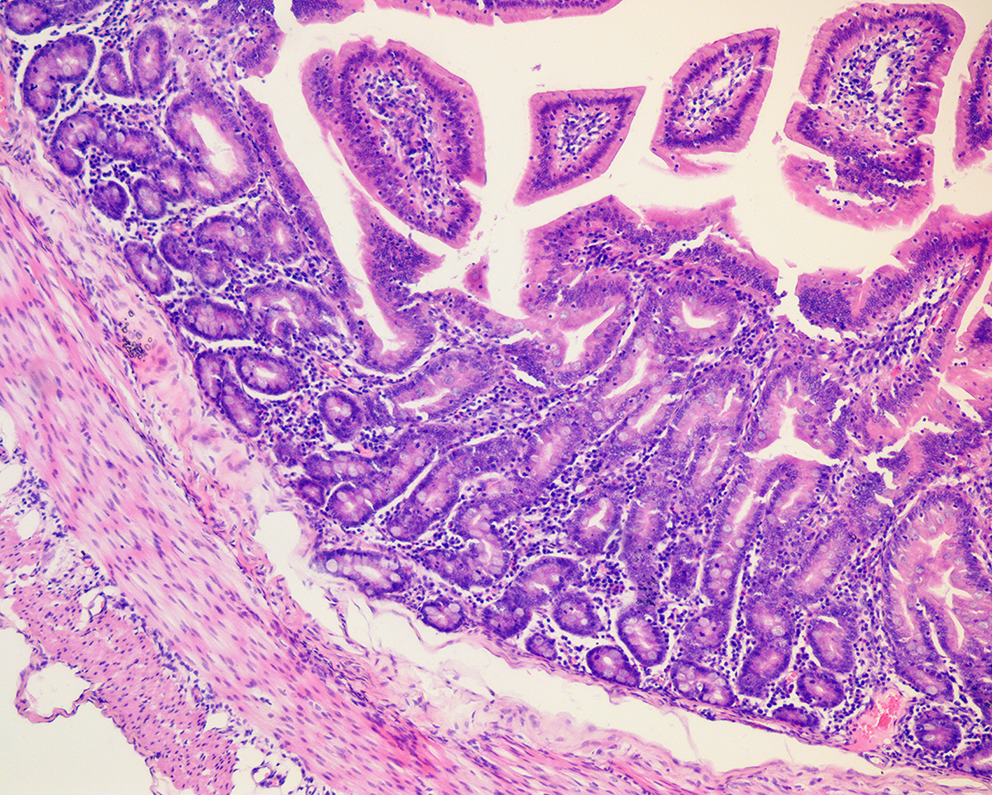

Supplement: Supplementary file 1 [file ijms-23-10395-s001.zip › ijms-1877568-supplementary/Original Date/Jejunum-CN-down.tif]

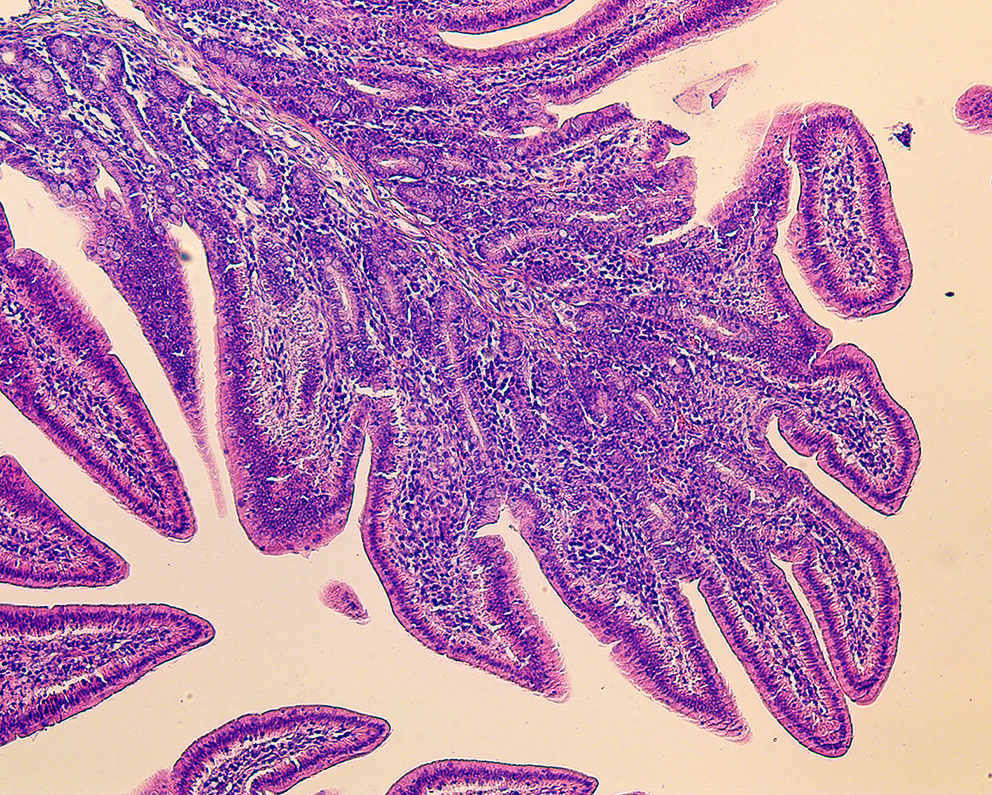

Supplement: Supplementary file 1 [file ijms-23-10395-s001.zip › ijms-1877568-supplementary/Original Date/Jejunum-CN-up.tif]

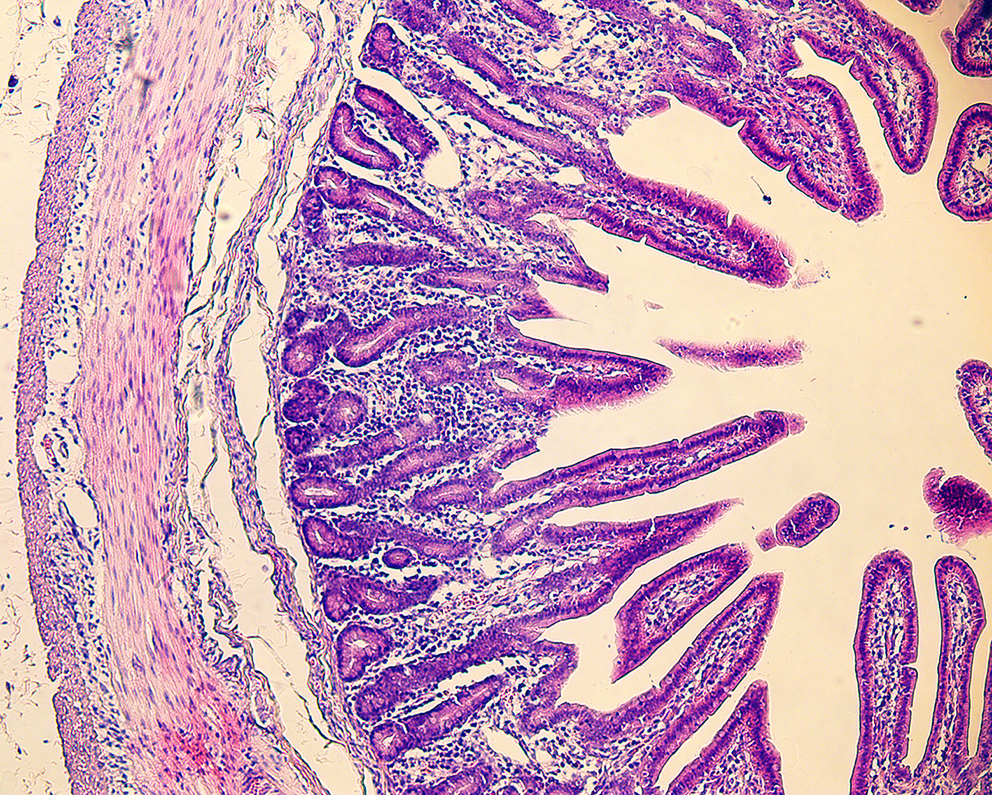

Supplement: Supplementary file 1 [file ijms-23-10395-s001.zip › ijms-1877568-supplementary/Original Date/Jejunum-LS-down.tif]

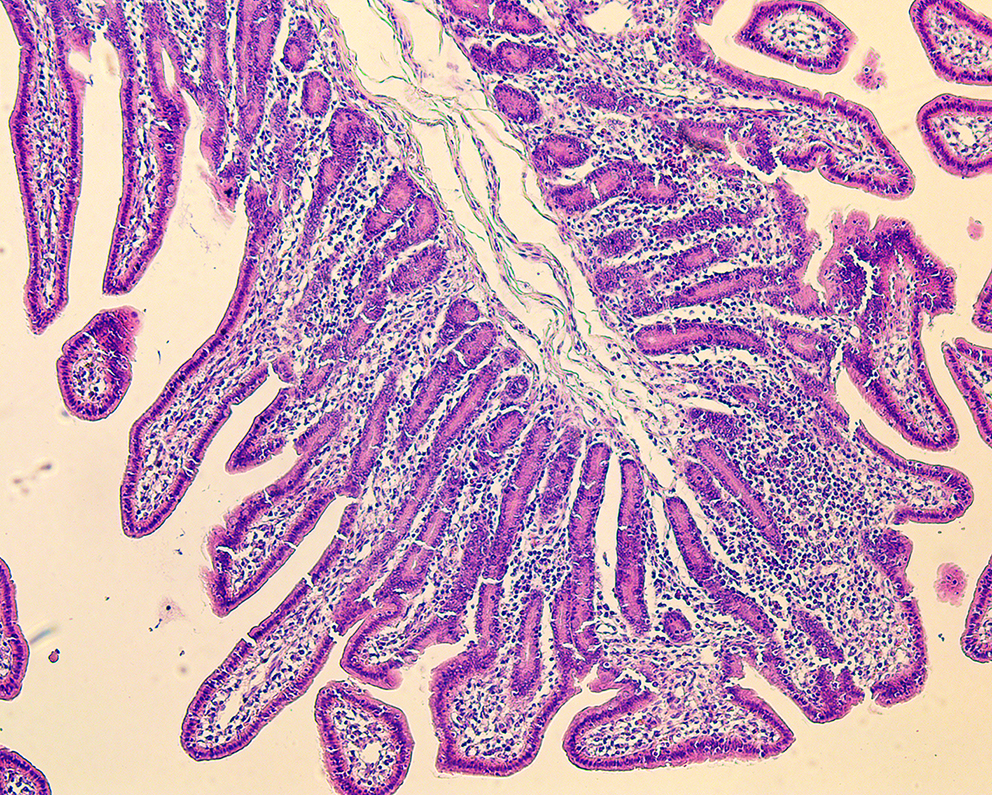

Supplement: Supplementary file 1 [file ijms-23-10395-s001.zip › ijms-1877568-supplementary/Original Date/Jejunum-LS-up.tif]

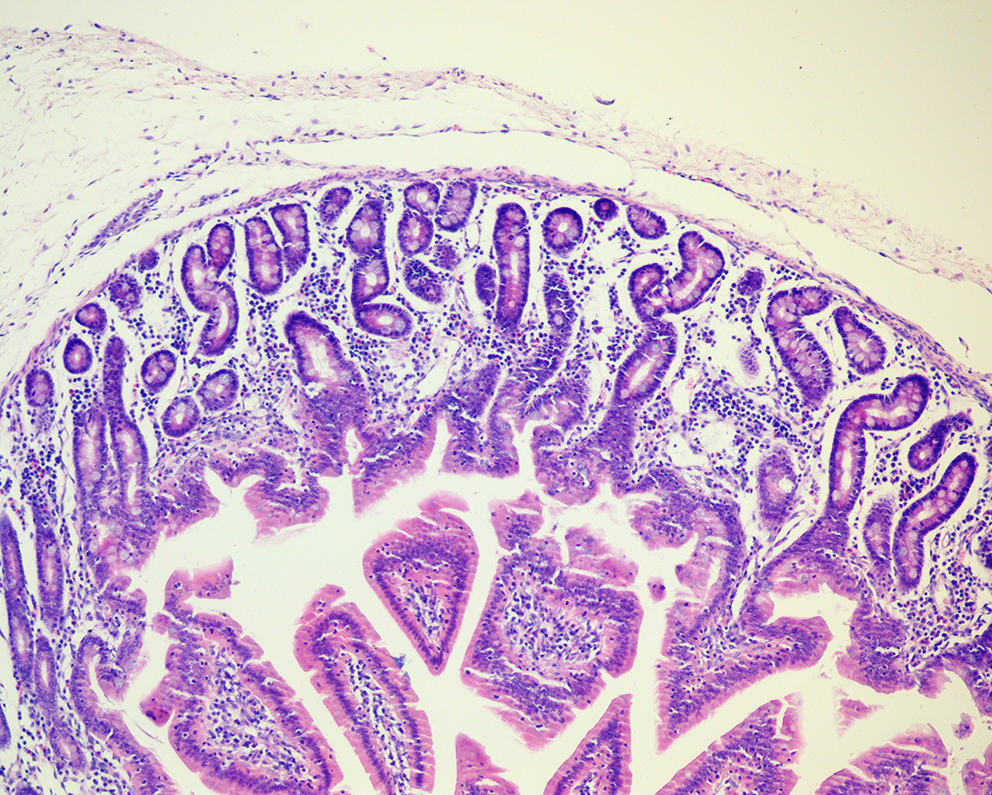

Supplement: Supplementary file 1 [file ijms-23-10395-s001.zip › ijms-1877568-supplementary/Original Date/Jejunum-SI-down.tif]

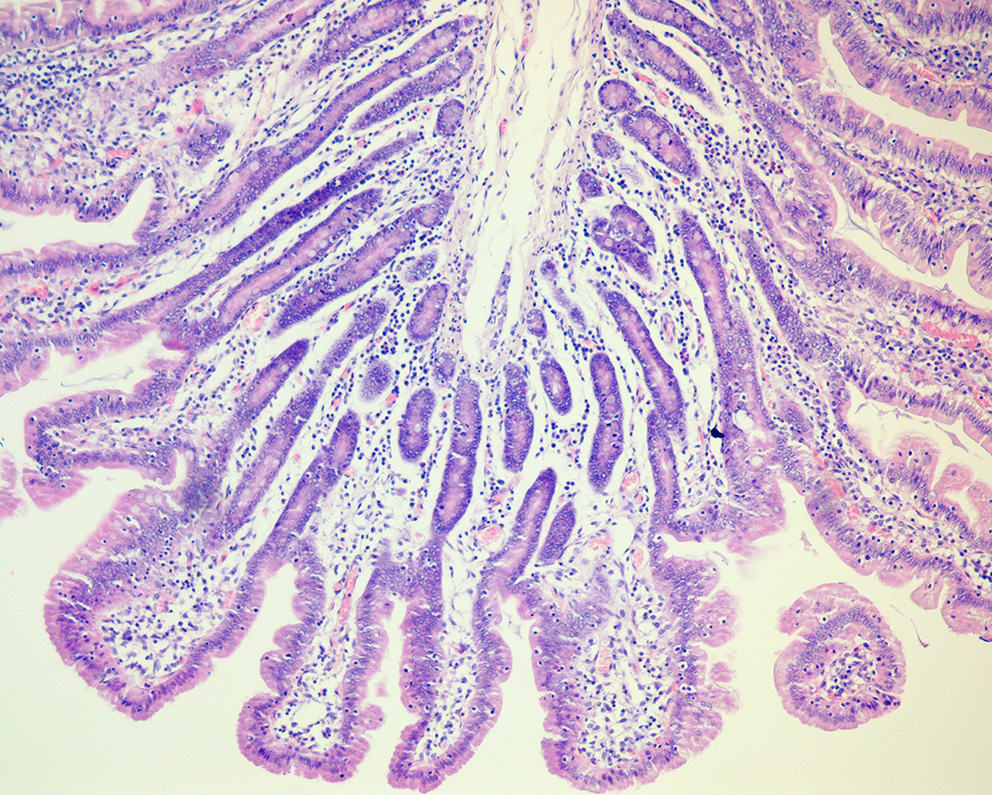

Supplement: Supplementary file 1 [file ijms-23-10395-s001.zip › ijms-1877568-supplementary/Original Date/Jejunum-SI-up.tif]

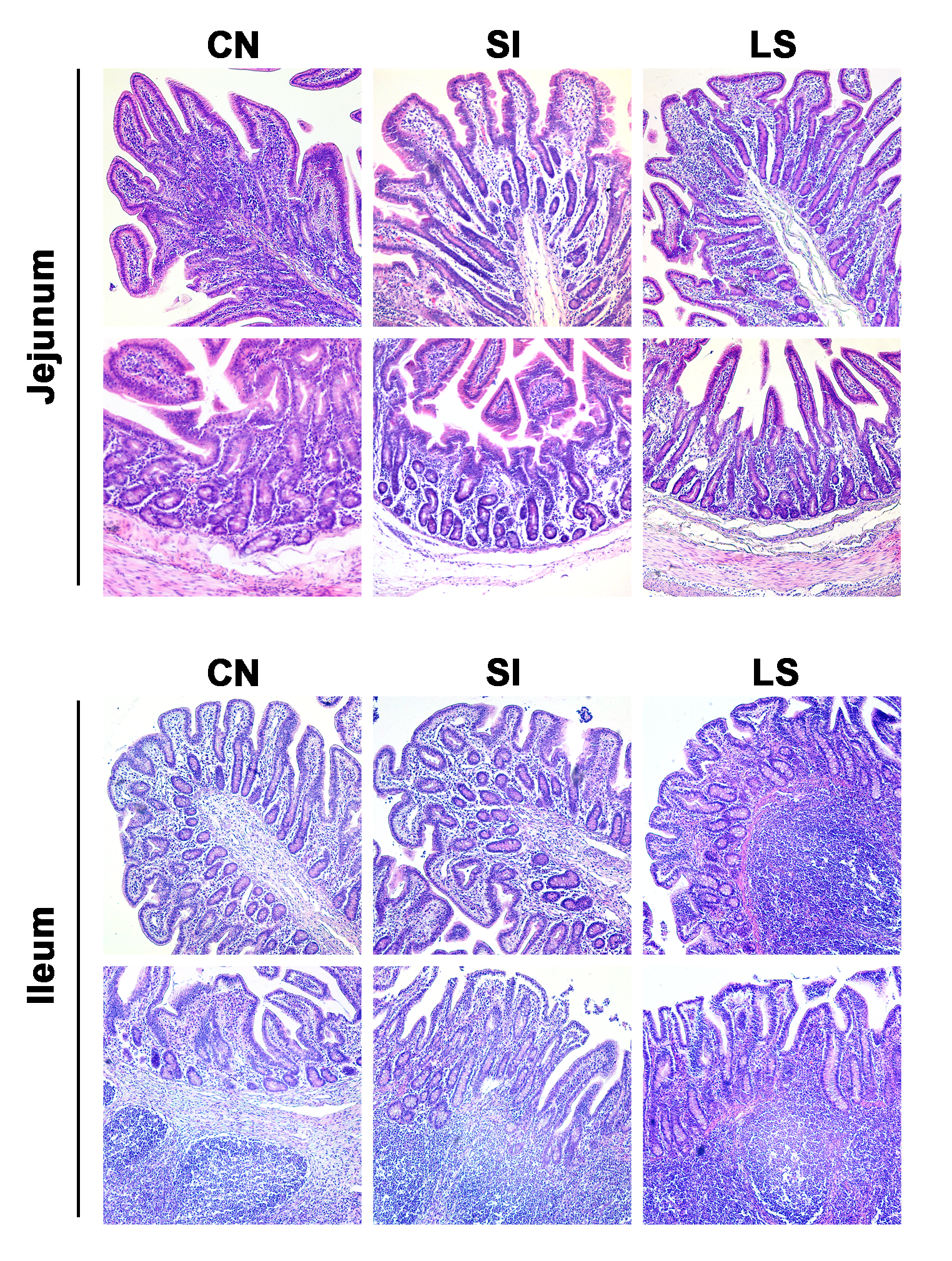

Supplement: Supplementary file 1 [file ijms-23-10395-s001.zip › ijms-1877568-supplementary/Supplementary Figure S1.tif]
